# Kinetics and thermodynamic studies for removal of acid blue 129 from aqueous solution by almond shell

**DOI:** 10.1186/2052-336X-12-62

**Published:** 2014-03-12

**Authors:** Mohammad Reza Fat’hi, Arash Asfaram, Anahita Hadipour, Mostafa Roosta

**Affiliations:** 1 Department of Chemistry, College of Science, Shahid Chamran University, P.O. Box: 6135743337, Ahvaz, Iran; 2 Young Researchers and Elite Club, Gachsaran Branch, Islamic Azad University, Gachsaran, Iran; 3 Department of Chemistry, Islamic Azad University, Gachsaran Branch, P.O. Box 75818-63875, Gachsaran, Iran; 4 Young Researchers and Elite Club, Sepidan Branch, Islamic Azad University, Sepidan, Iran

**Keywords:** Acid blue 129, Almond shell, Equilibrium studies, Kinetics, Thermodynamics

## Abstract

Efficiency and performance of Almond shell (AS) adsorbent for the removal and recovery of Acid Blue 129 (AB129) from wastewater is presented in this report. The influence of variables including pH, initial dye concentration, adsorbent dosage, particle size, contact time and temperature on the dye removal have been investigated in batch method by one at a time optimization method. The experimental equilibrium data were tested by four widely used isotherm models namely, Langmuir, Freundlich, Tempkin and Dubinin-Radushkevich (D–R). It was found that adsorption of AB129 on AS well with the Langmuir isotherm model, implying monolayer coverage of dye molecules onto the surface of the adsorbent. More than 98% removal efficiency was obtained within 14 min at adsorbent dose of 0.4 g for initial dye concentration of 40 mg/L at pH 2. Kinetics of the adsorption process was tested by pseudo-first-order and pseudo-second-order kinetics, and intraparticle diffusion mechanism. Pseudo-second-order kinetic model provided a better correlation for the experimental data studied in comparison to the pseudo-first-order model. Calculation of various thermodynamic parameters such as, Gibb’s free energy, entropy and enthalpy of the on-going adsorption process indicate feasibility and endothermic nature of AB129 adsorption on all adsorbents. This work can be used in design of adsorption columns for dyes removal.

## Introduction

Textile industry uses large volumes of water in wet processing operations and thereby, generates large amounts of dissolved dyestuffs and other products such as dispersing agents, dye bath carriers, salts, emulsifiers, leveling agents and heavy metals [1]. Colored dyes are not only aesthetic, carcinogenic but also hinder light penetration and disturb life processes of living organisms in water. Acid Blue 129 (AB129), an acidic dye, is most widely used for the dyeing of cotton, wool, silk, nylon, paper and leather (Table 1). This dye may be harmful if contact to eyes, respiratory system and skin. Therefore, the removal of such colored agents from aqueous effluents is necessary [2].

**Table 1:** Properties of the acid blue 129

**C.I. number**	**Acid blue 129**
**Chemical formula**	C_23_H_19_N_2_ NaO_5_S
**Molecular weight**	458.46
**Name**	Sodiume-1-amino-4-(2, 4, 6-trimethylanilino) anthraquinone-2-sulfonate.
**Maximum wavelength (nm)**	600

Traditionally, well known protocols such as coagulation, nano filtration and ozonalysis, membrane filtration, oxidation and adsorption process are applied to remove color and other contaminations from aqueous media [3-7]. Adsorption is most popular technique that benefit from advantages such as high efficiency and ability to use generable non-toxic and cheap adsorbents [8-22]. Although, activated carbon appears to be the widely used techniques for dye removal, but in view of the high cost and regeneration problems scope of many adsorption studies has been focused to derive cheaper adsorbents from the waste materials [23]. During the past decades, several researches are to utilize low cost and easily available natural materials as potential adsorbents for removal of dyes [24-29]. The present work aims to study a convenient and economic method for AB129 removal from water by adsorption on AS as low cost and abundantly available adsorbent. The effects of initial AB129 concentration, contact time, pH, particle size and amount of adsorbent on AB129 removal have been evaluated.

## Materials and methods

### Chemicals and instruments

AS were supplied from the Arctic Sea Region of Iran. They were firstly dried, crushed in a ball mill and sieved to obtain a particle size between 0–177, 210–297 and 350–500 μm. All chemical used were of analytical grade and doubled distilled water was used throughout. A stock solution of 200 mg/L of AB129 was prepared by dissolving 0.100 g of solid dye in water and diluting to 500 mL in a volumetric flask. The AB129 concentration evaluation was carried out using Shimadzu UV–vis spectrophotometer model 160A (Shimadzu, Japan) at a wavelength of 600 nm. The pH measurements were carried out using pH/Ion meter model 691 (Metrohm, Switzerland, Swiss).

### Method

To study the effect of important parameters like the pH, adsorbent dosage, contact time, initial dye concentration and temperature on the adsorptive removal of AB129 batch experiments were conducted. For each experimental run, 25 mL of AB129 solution of known concentration, pH and amount of the adsorbent were taken in a 50 ml Erlenmeyer flask with middle magnet. This mixture was agitated on stirrer at a constant speed in a temperature controlled. Samples were withdrawn at different time intervals (0–15 min for AS) and kinetics, thermodynamic, isotherm and other parameters of adsorption was determined by analyzing of remaining dye concentration from aqueous solution.

Experiments were carried out at pH = 2.0 that the initial pH of the solution was adjusted by addition of aqueous solutions of HCl or NaOH.

For adsorption isotherms, dye solutions of different concentrations (10–90 mg/L) and at temperatures (25°C) were agitated with known amounts of adsorbents until the equilibrium was achieved.

## Results and discussion

**Figure 1:**
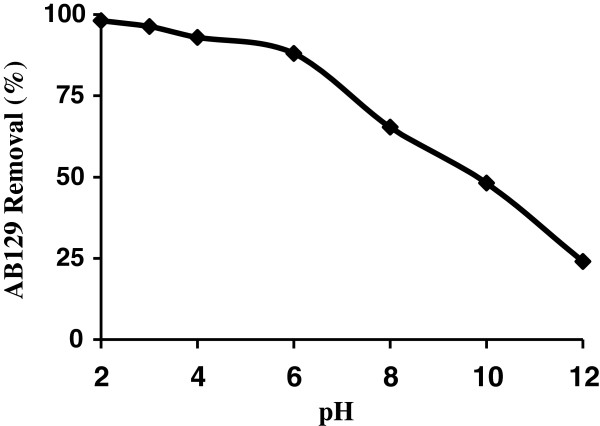
The influence of initial pH on AB129 sorbed on AS (AB129 concentration: 40 mg/L; sorbent dose: 0.4 g; contact time: 14 min).

Solution pH affects both aqueous chemistry and surface binding sites of the adsorbents. The effect of initial pH on adsorption of AB129 was studied from pH 2 to 12 at 25°C, initial dye concentration of 40 mg/L, adsorbent dosage of 0.4 g and contact time of 10 min. The maximum adsorption of the AB129 is obtained at pH = 2 (Figure 1). The acidity constant value of the most acidic group of the AB 129 molecule is 1.6. This functional group can be easily dissociated and thus, the AB 129 molecule has net negative charges in the working experimental conditions [30,31].

At acidic pH the H^+^ ion concentration in the system increased and the surface of the AS acquires positive charge by absorbing H^+^ ions. As the pH of the system increases, the number of negatively charged sites increases and the number of positively charged sites decreases. Negatively charged surface sites on the AS do not favor the adsorption of AB129 anions due to the electrostatic repulsion. Also lower adsorption of AB129 at alkaline pH is due to the presence of excess OH¯ ions, which destabilize anionic AB129 and compete with the AB129 anions for the adsorption sites. The most effective pH was 2.0 and it was used in further studies.

**Figure 2:**
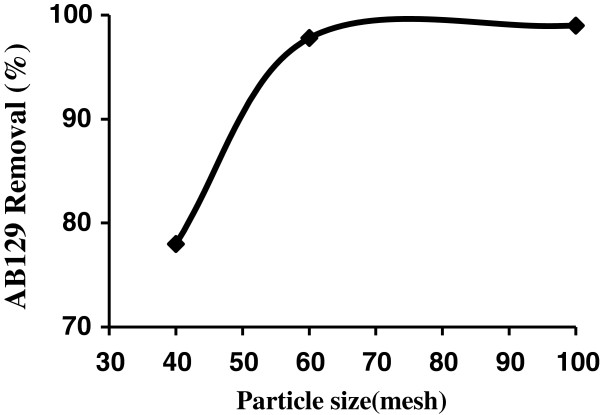
The influence of particle size on AB129 sorbed on AS (AB129 concentration: 40 mg/L; sorbent dose: 0.4 g/25 ml; contact time: 14 min).

The particle size distribution of AS determined by sieving the samples manually shaking with stainless steel mesh screens of standard (international ASTM with meshes 40, 60 and 100). For batch adsorption experiments, three different particle sizes viz. 40 and 60–100 AS mesh were selected and difference in the amount adsorbed was noticed by using different mesh sizes. Effect of sieve size of adsorbent on the adsorption was studied at 25°C, 0.4 g of AS, pH = 2 and 40 mg/L of AB129. It was observed that adsorption was found to increase with the 60–100 mesh sizes in Figure 2. This is due to increase in the surface area of the adsorbent and accessibility of the adsorbent pores towards the AB129 [32].

**Figure 3:**
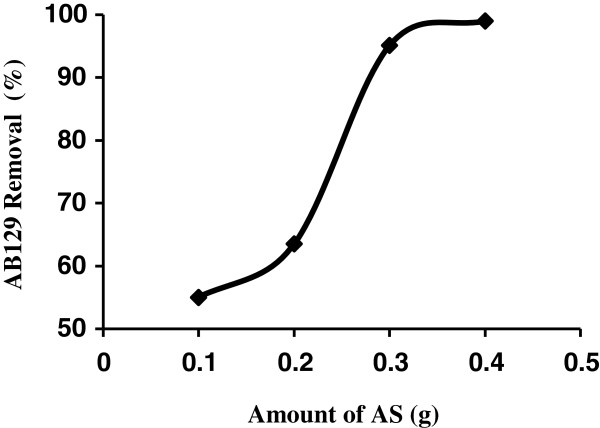
Effect of sorbent dose on AB129 sorbed on AS (AB129 concentration: 40 mg/L; sorbent dose: 0.4 g; contact time: 14 min.

Figure 3 demonstrates the effect of adsorbent dosages for removal of the AB129 from aqueous solution. It was observed that highest amount of AB129 removal was attained for adsorbent mass of at least adsorbent.

For adsorbent dosage higher than 0.4 g the AB129 removal remained almost constant. Increases in the percentage of AB129 removal with adsorbent dosage could be attributed to increases in the adsorbent surface areas, augmenting its number of adsorption sites available for adsorption [33,34]. In order to continue this work, the adsorbent dosage was fixed at 0.4 g, since this adsorbent dosage correspond to the minimum amount of adsorbent which lead to a constant and maximum removal of AB129.

**Figure 4:**
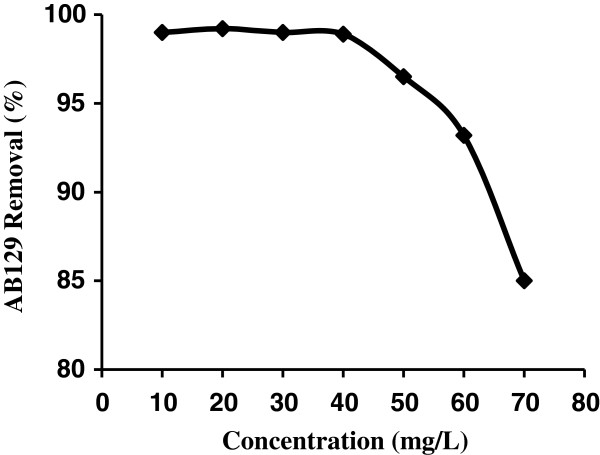
The effect of AB129 concentration on sorbed on AS (sorbent dose: 0.4 g/25 mL; contact time: 14 min).

Studies for investigation of the effect of dye concentration on its removal carried out by adding of 0.4 g prepared AS in 25 mL of solution at different concentration of 10–70 mg/L, pH = 2 and room temperature (25°C). As shown in Figure 4, by increasing of initial dye concentration, removal of dye was decreased.

**Figure 5:**
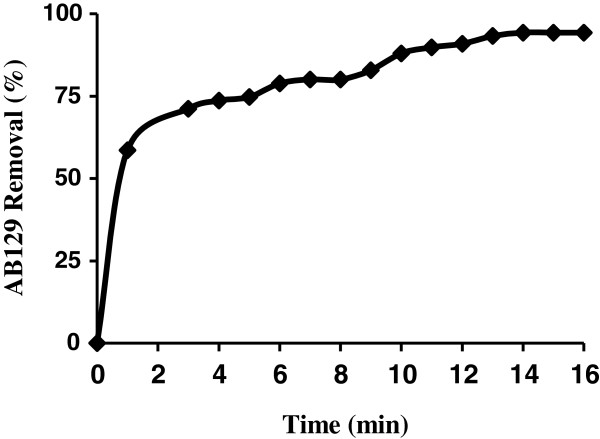
The effect of contact time on AB129 sorbed on AS (AB129 concentration: 40 mg/L; sorbent dose: 0.4 g; pH: 2).

The adsorption rate, obtained for AB129 adsorption on AS was observed by decrease of the concentration of AB129 within the adsorption medium with contact time. As shown in Figure 5, it can be concluded that maximum dye removal could be achieved when the sonication time was above 14 min. After equilibrium, the amount of adsorbed dye did not change significantly with time.

At 40 mg/L of AB129, the removal rate in the first varies from 58.5% to 94.24% of the maximum removal onto AS. For instance, the adsorbents exhibited three stages, which can be attributed to each linear portion of the figure. The first linear portion was attributed to the diffusion process of AB129 to the adsorbent surfaces [35,36], hence, was the fastest adsorption stage.

This result is corroborated by the factionary-order kinetic model. The second linear portion was attributed to intra-particle diffusion, which was delayed process. The third stage may be regarded as the diffusion through smaller pores, which is followed by the establishment of equilibrium. The surface of AS may contain a large number of active sites and the solute adsorption can be related to the active sites on equilibrium time. Also up to 90% of the total amount of AB129 adsorption was found to occur in the first rapid phase (10 min) and thereafter the adsorption rate was found to decrease. The higher adsorption rate at the initial period (first 10 min) may be due to too number of vacant sites available at the initial stage. As a result there exist too concentration gradients between adsorbate in solution and onto adsorbent surface. This increased in concentration gradients tends to increase in AB129 adsorption at the initial stages.

### Adsorption kinetic studies

The mechanism of adsorption was investigated by pseudo first order and pseudo second order models.

**Figure 6:**
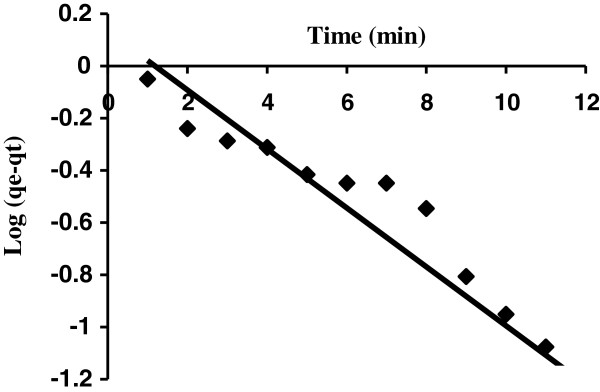
Pseudo-first-order kinetic model plot for the adsorption on AS (AB129 concentration: 40 mg/L; sorbent dose: 0.4 g/25 mL; contact time: 14 min).

Based on the pseudo first-order expression (Lagergren model) by plotting the values of log (*q*_*e*_ − *q*_*t*_) against *t* give a linear relationship that *q*_*e*_ and *k*_1_values can be determined from the intercept and slope of the obtained line, respectively (Figure 6 and Table 2). The parameters qe and qt are amounts of dye adsorbed (mg/g) on adsorbent at equilibrium and at time t, respectively and k1 is rate constant of pseudo first order adsorption (1/min).

**Table 2:** Kinetic parameters for the adsorption of 40 mg/L AB129 onto AS

**Model**	**Equation**	**Parameters**	**Value**
**Pseudo-first-order kinetic**	Log (q_e_ ‐ q_t_) = log(q_e_) ‐ k_1_/2.303t	k_1_(1/min)	0.210
		q_e_ (calc) (mg/g)	1.096
		R^2^	0.912
**Pseudo-second-order kinetic**	(t/q_t_) = 1/(k_2_q_e_^2^) + 1/q_e_(t)	k_2_ (g/(mg min))	0.313
		q_e_ (calc) (mg/g)	2.532
		R^2^	0.994
		H (mg/(g min))	1.731
**Intraparticle diffusion**	q_t_ = K_dif_t^1/2^ + C	K_diff_ (mg/(g min^1/2^))	0.519
		C (mg/g)	1.221
		R^2^	0.949
**Elovich**	q_t_ = 1/β ln(αβ) + 1/β ln(t)	β (g/mg)	3.068
		α (mg/(g min))	10.21
		R^2^	0.973
		q_e_ (exp) (mg/g)	2.356

Figure 6 was used to determine pseudo first order rate constant (k1) and theoretical amount of dye adsorbed per unit mass of adsorbent qe(the). Distance of qe(the) from qe(exp) value indicate that this model was not fit well with the experimental data (Table 2) [37].

**Figure 7:**
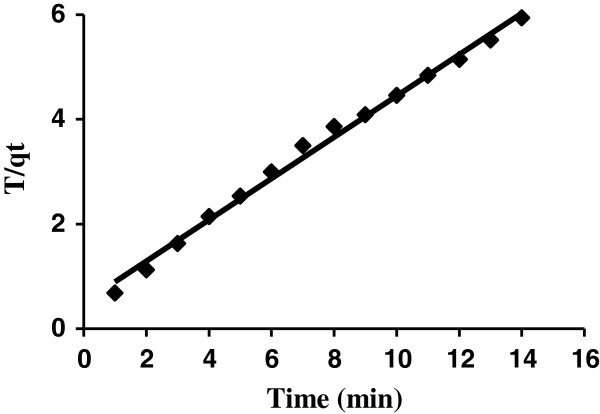
Pseudo-second-order kinetic model plot (AB129 concentration: 40 mg/L; sorbent dose: 0.4 g/25 mL; contact time: 14 min).

The plot of *t*/*q*_*t*_ versus *t* for the pseudo-second-order kinetic model gives a straight line that *k*_2_ and equilibrium adsorption capacity (*q*_*e*_) were calculated from the intercept and slope of this line, respectively (Figure 7). Where k2 is rate constant of second order adsorption (g/(mg min)). The high value of R^2^ (0.994) and closeness of experimental and theoretical adsorption capacity (qe) value show the applicability of this model to explain the experimental data (Table 2).

### Adsorption equilibrium study

Adsorption isotherms are prerequisites to understand the nature of the interaction between adsorbate and the adsorbent used for the removal of organic pollutants [38,39]. The equation parameters of various adsorption isotherm equations such as Langmuir, Freundlich, Temkin, Dubinin-Radushkevich (D-R) and Harkins-Jura isotherms provide some insight into the adsorption mechanism, the surface properties and affinity of the adsorbent for adsorbate [40,41].

The Langmuir isotherm is based on the assumption that the adsorption process takes place at specific homogeneous sites within the adsorbent surface and that once a dye molecule occupies a site, no further adsorption can take place at that site, which concluded that the adsorption process is monolayer in nature.

**Figure 8:**
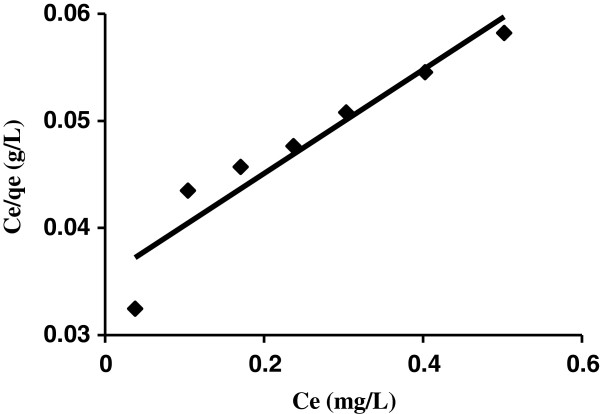
Langmuir isotherm models plot for the adsorption on AS (AB129 concentration: 10–90 mg/L; sorbent dose: 0.4 g/25 ml; contact time: 14 min).

Langmuir isotherm is based on the assumption that:

a) Maximum adsorption corresponds to a saturated monolayer of adsorbate molecules on the adsorbate surface.

b) The energy of the adsorption is constant.

c) There is no transmigration of adsorbate molecules in the plane of adsorbent surface [42]. Based on the linear form of Langmuir isotherm model (according to Table 3), the values of *K*_*a*_ (the Langmuir adsorption constant (L/mg)) and *Q*_*m*_ (theoretical maximum adsorption capacity (mg/g)) were obtained from the intercept and slope of the plot of C_e_/q_e_ vs C_e_, respectively (Figure 8). The values of Qm and *K*_*a*_ are 11.95 m/g and 0.902 L/mg, respectively shown in Table 3. The high correlation coefficient (0.994) shows the applicability of Langmuir model for interpretation of the experimental data.

**Table 3:** Isotherm constant parameters and correlation coefficients calculated for the adsorption of AB129 onto AS

**Isotherm**	**Equation**	**Parameters**	**Value**
**Langmuir**	1/q_e_ = 1/(K_a_Q_m_C_e_) + 1/Q_m_	Qm (mg/g)	11.95
		Ka (L/mg)	0.902
		R2	0.994
**Freundlich**	ln q_e_ = ln K_F_ + (1/n)ln C_e_	1/n	0.508
		KF (L/mg)	4.779
		R2	0.929
**Tempkin**	q_e_ = B_1_ ln K_T_ + B_1_ ln C_e_	B1	2.373
		KT (L/mg)	11.35
		R2	0.993
**Dubinin-Radushkevich**	ln q_e_ = ln Q_s_ ‐ Bε^2^	Qs (mg/g)	8.207
		B × 10-5	0.8
		E	250
		R2	0.948

The data was analyzed by the linearized form of Freundlich isotherm model. In this model, q_e_ is the amount of adsorption, kf is the Freundlich constant related to sorption capacity and 1/n is a constant related to energy or intensity of adsorption. This gives an expression encompassing the surface heterogeneity and the exponential distribution of activated sites and their energies. This isotherm dose not predicts any saturation of the adsorbent surface. The Freundlich exponents k_F_ and 1/n can be determined from the linear plot of log qe *vs.* log Ce is shown in Table 3. The values of the Freundlich constants K_F_ and 1/n are 0.508 respectively shown in Table 3. The slope 1/n ranging between 0 and 1 is a measure of adsorption intensity or surface heterogeneous, becoming more heterogeneous as its value gets closer to zero [43].

Heat of adsorption and the adsorbent-adsorbate interaction on adsorption isotherms were studied by Tempkin [44]. The constants obtained for Tempkin isotherm are shown in Table 3. The linear form of Dubinin-Radushkevich isotherm equation was applied to estimate the porosity apparent free energy and the characteristic of adsorption [45]. The constant obtained for D–R isotherms are shown in Table 3. The mean adsorption energy (*E*) gives information about chemical and physical nature of adsorption.

As seen from Table 3, the Langmuir model yields a somewhat better fit (R^2^ = 0.994), Tempkin isotherm (R^2^ = 0.993) than the Freundlich model (R^2^ = 0.929) and Dubinin-Radushkevich model (R^2^ = 0.948). Equilibrium data fitted well with the Langmuir and Tempkin model.

### Thermodynamic study

Thermodynamic parameters such as change in free energy (∆G°) (J/mole), enthalpy (∆H°) (J/mole) and entropy (∆S°) (J/(K mole)) were determined using following equations

(1) $$K_{o}=\mathrm{Csolid}/\mathrm{Cliquid}$$

(2) $$\begin{array}{l} \mathrm{∆G}=‐\mathrm{RT}\;{\mathrm{lnK}}_{o}\\ \mathrm{∆G}=\mathrm{∆H}‐\mathrm{T∆S}\\ {\mathrm{lnK}}_{o}=‐\mathrm{∆G}/\mathrm{RT} \end{array}$$

(3) $${\mathrm{lnK}}_{o}=∆S/R‐∆H/\mathrm{RT}$$

**Figure 9:**
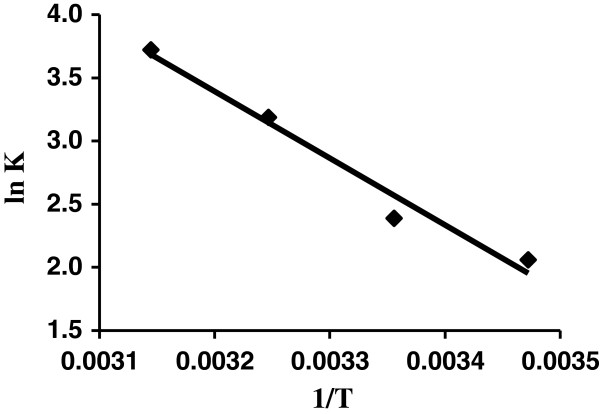
Van’t Hoff plots for the adsorption of AB129 (40 mg/L) onto AS for evaluating thermodynamic parameters.

Where K_o_ is equilibrium constant, C_solid_ is solid phase concentration at equilibrium (mg/L), C_liquid_ is liquid phase concentration at equilibrium (mg/L), T is absolute temperature in Kelvin and R is gas constant. ∆G° values obtained from equation (2), ∆H° and ∆S values obtained from the slope and intercept of plot lnK_o_ against 1/T (Figure 9). The negative value of ∆G° indicates the adsorption is favorable and spontaneous (Table 4). ∆*H*◦ and ∆*G*◦ can be obtained from the slope and intercept of Van’t Hoff plot of ln *K*c vs. 1/*T*.

**Table 4:** Thermodynamic parameters for adsorption of AB129

**Adsorbent**	**C**_**0**_**(mg/L)**	**Parameter**	**Temperature (K)**			
			288	298	308	318
**Apricot Stone (AS)**	40	**k**_**C**_	7.845	10.888	24.24	41.33
		**∆G° (** kj/mol**)**	-4.93	-5.91	−8.16	-9.83
**C**_**0**_**(mg/L)**		**∆S°** (J/(mol k))			**∆H°** (kj/mol)	
40		168.74			43.91	

The positive values of ∆H◦ further confirm the endothermic nature of the adsorption process and the positive ∆S◦ values suggest the increase in adsorbate concentration in solid–liquid interface indicating thereby the increase in adsorbate concentration onto the solid phase. It also confirms the increased randomness at the solid–liquid interface during adsorption. This is the normal consequence of the physical adsorption phenomenon, which takes place through electrostatic interactions.

### Comparison with literature

The performance of the proposed method has been compared with other adsorbents. As is seen in Table 5 the contact time for proposed method in comparison with all of the adsorbents are preferable and superior to the literature which show satisfactory removal performance for AB129 as compared to other reported adsorbents [46,47].

**Table 5:** Comparison for the removal of AB129 by different adsorbents

**Adsorbent**	**Adsorbate**	**Contact time (min)**	**Ref**
activated carbon cloth	AB129	400	[46]
Row bentonite	AB129	>1000	[47]
CTAB-bentonite	AB129	>100	[47]
Almond shell	AB129	14	This work

## Conclusion

It was observed that the AS is an efficient adsorbent for the removal of AB129S. Removal of AB129 is pH dependent and the maximum removal was attained at pH = 2. The equilibrium data fitted very well in a Langmuir and Tempkin isotherm equations. The data indicate that the adsorption kinetics follow the pseudo-second-order with spontaneous and endothermic nature of the adsorption process. The positive sign of ∆S° indicates that the adsorption process takes place through electrostatic interaction between adsorbent surface and adsorbate species in solution. The present study concludes that the AS could be employed as low-cost adsorbents for the removal of AB129 from aqueous solution in general.

## Competing interests

The authors declare that they have no competing interests.

## Authors’ contributions

All authors read and approved the final manuscript.
